# An investigation into General Practitioners’ experience with Long Covid

**DOI:** 10.1007/s11845-024-03782-7

**Published:** 2024-08-20

**Authors:** Aisling Farrell, James O’Flynn, Aisling Jennings

**Affiliations:** 1 University College Cork, Cork, Ireland; 2 Department of General Practice, University College Cork, Cork, Ireland

**Keywords:** Covid, General practitioner, Long Covid, Patient care

## Abstract

**Background:**

Long Covid (LC) is the continuation or development of new symptoms after initial COVID-19 infection. Little is known about General Practitioners’ (GP) experience of managing patients with LC.

**Aims:**

The aim of this study is to establish GP experiences with LC.

**Methods:**

A survey was designed and piloted in three training practices prior to distribution. The survey was completed by doctors working in GP training scheme practices in Cork, Ireland.

**Results:**

Fifty-three of one hundred and sixty invited GPs completed the survey, indicating a 33% response rate. 8% (4/53) of participants agreed and 0% (0/53) strongly agreed with the statement they were “confident in diagnosing Long Covid”. 81% (43/53) were not confident in treating patients with LC. 70% (37/53) were unaware of indications for referral to secondary care. 38% (20/53) were aware of the referral pathways to local LC clinics. 93% (49/53) agreed there were educational deficits regarding LC.

**Conclusions:**

There was a lack of confidence in the diagnosis and management of LC, and in the interface with secondary care. There is demand for educational interventions to assist GPs with their care of patients with this emerging condition.

**Supplementary Information:**

The online version contains supplementary material available at 10.1007/s11845-024-03782-7.

## Introduction

Long Covid (LC) is defined as the continuation or development of new symptoms three months after initial Covid-19 infection [1]. Four common phenotypes of LC have been described in the literature including chronic fatigue–like syndrome, respiratory syndrome neurosensorial syndrome and chronic pain syndrome [2, 3]. The prevalence of LC in Ireland remains under investigation [4]. LC is a multifaceted condition requiring a holistic approach and ongoing support, thus General Practitioners (GPs) are well suited to care for patients with this condition [5].

The Covid pandemic placed unprecedented demand on healthcare systems globally. GPs played a central role during the pandemic as they adapted to a constantly evolving situation [6, 7]. As the ‘secondary epidemic’ of LC emerges, it has been demonstrated internationally that GPs now frequently see patients with this condition [8]. GPs are faced with further challenges as they navigate the uncertainty surrounding treating these patients [9]. Given the heterogenicity of LC symptoms encountered by GPs, it is likely that this will contribute to an increase in service demands [8].

Evidence to inform the management of LC is sparse in the literature and GP decisions are often made under a great deal of uncertainty [3, 9]. Available guidance suggests a focused history and examination to guide initial assessment and help out-rule alternative diagnoses [3, 10]. Self-management of symptoms with continued GP support is first line management [11]. There is insufficient evidence for specific pharmacological intervention [3]. Patients with persistent, debilitating symptoms should be referred for specialist assessment; however, specific indications for referral to LC clinics are not clear [3, 12]. Additionally, inadequate access to tertiary services for patients has been demonstrated internationally [12].

A number of studies have investigated patient experiences with LC and the experiences of doctors who are themselves suffering with LC [13–16]. However, despite the key role GPs play in the management of the condition, there is a paucity of data exploring GP experiences of managing LC [17]. A small number of international studies have looked specifically at GP experiences with LC but these studies have mostly focused on symptoms encountered and treatments provided [18–21]. A broader exploration into the perspectives and challenges GP face when diagnosing and managing LC is lacking, particularly in an Irish healthcare setting [12].

The aim of this study is therefore to establish GPs experiences with LC across the domains of diagnosis, management, education, and service-related impacts. This research intends to reveal targets for future interventions aimed at providing better support to GPs and thus improving care for patients with LC.

## Methods

This was a cross-sectional study of GP experiences with LC in the Republic of Ireland. A survey tool was designed to extract demographic data as well as a broad overview of the GP experience of and confidence with LC across the key areas of relevance to GPs including assessment, management, secondary care services and education [11]. This survey was designed and piloted in three GP practices prior to distribution. The study protocol was reviewed and approved by the Irish College of General Practitioner’s Research Ethics Committee.

The study participants were practising GPs and GP Registrars working within a network of GP training practices in southern Ireland. We estimated our sample size to be 160 and aimed for a 50% response rate given the novel nature of this condition. Participants were invited to participate via an email containing a link to the survey. The online survey was held on Google forms and was available for a one month period in September 2023 (see Supplementary material A).

Using a combination of forced choice items, free text boxes and five-point Likert scales, questions examined GP’s experiences of managing patients with LC. Descriptive statistics were used to analyse the data.

## Results

The survey data was collected between September and October 2023. Of 160 people contacted, 53 responded (response rate 33%). Demographic data is represented in Table 1.

**Table 1:** Characteristics of participants, *n* = 53

	*n* (%)
Qualification	
GP Registrar	26 (49)
Qualified GP	27 (51)
Years experience	
0–5	24 (45)
5–10	12 (23)
10 +	17 (32)
Location of work	
Urban	19 (36)
Rural	16 (30)
Mixed	18 (34)

49.1% (26/53) of respondents reported that LC is relevant to their daily practice. 56.6% (30/53) and 11.3% (6/53) reported having diagnosed and managed LC in adult and in paediatric patients, respectively.

### Assessment and diagnosis

**Fig. 1:**
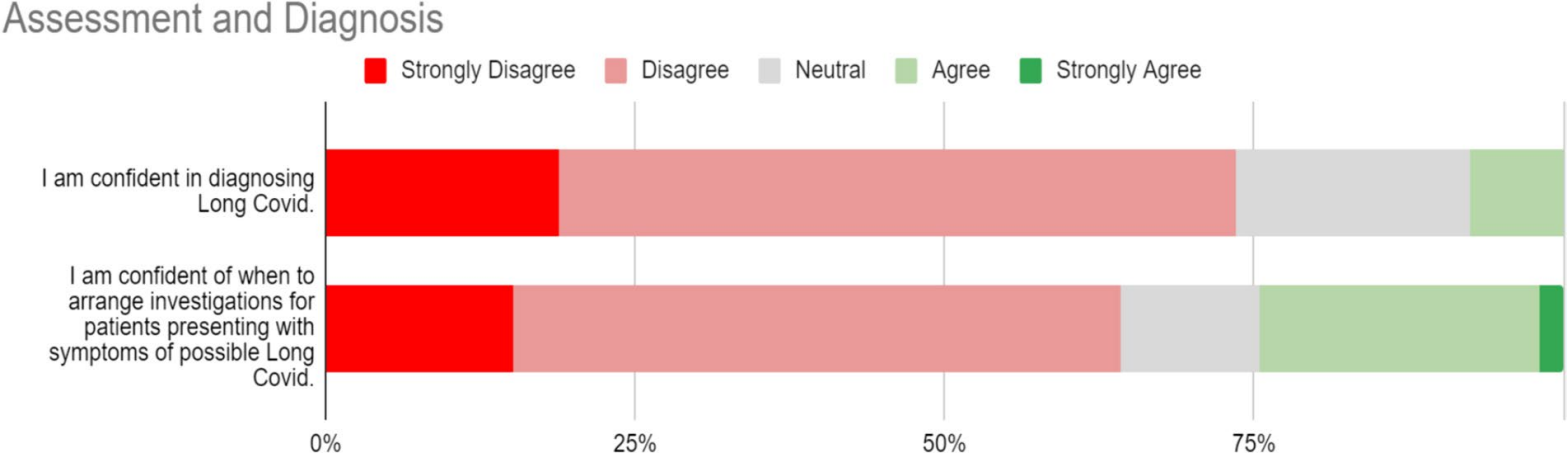
Assessment and diagnosis of Long Covid

Responses to statements regarding Assessment and Diagnosis of LC are represented in Fig. 1. No respondents strongly agreed with the statement “I am confident in diagnosing Long Covid”, and just 7.55% (4/53) agreed.

The majority of participants (64% (34/53)) also either strongly disagreed or disagreed with the statement ‘I am confident of when to arrange investigations for patients presenting with symptoms of possible Long Covid.” Only 1 participant strongly agreed with this statement. Only 17% (9/53) of respondents reported they use certain symptom criteria to diagnose LC. The most common symptom criteria nominated in free text responses were fatigue, respiratory symptoms, neurocognitive symptoms, and cardiovascular symptoms.

### Clinical management

**Fig. 2:**
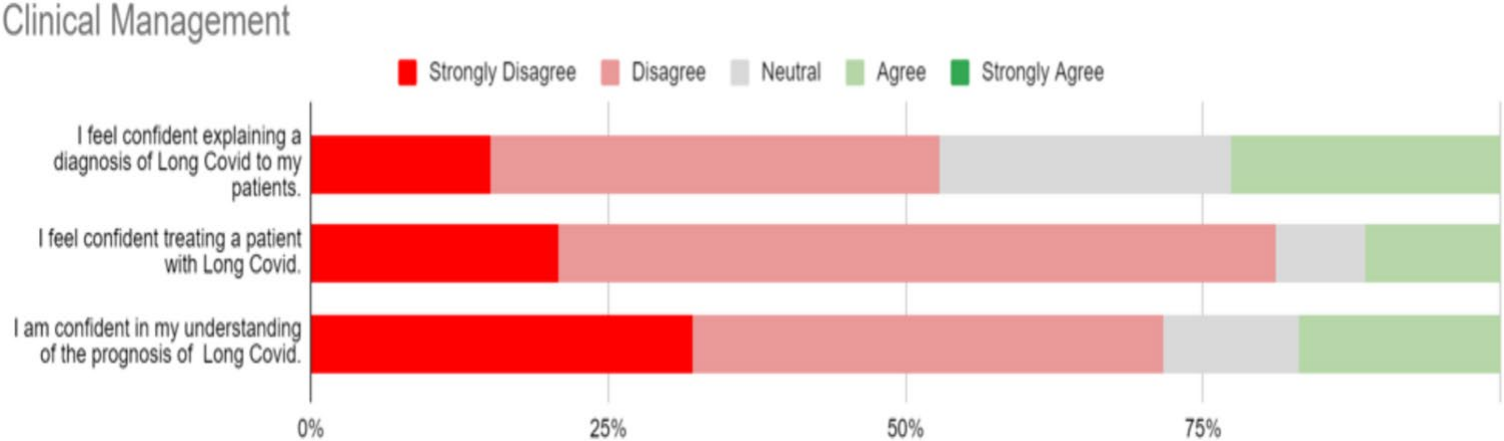
Clinical management of Long Covid

Responses to statements regarding the Clinical Management of LC are represented in Fig. 2. A statement of confidence in the explanation of LC to patients generated 52.83% (28/53) disagree or strongly disagree responses, and no strong agreement. 81.13% (43/53) of respondents strongly disagreed or disagreed that they were confident treating patients with LC. 71.70% (38/53) of the respondents strongly disagreed or disagreed with a statement of confidence in understanding the prognosis of the condition. 58.5% of respondents felt that a “Long Covid” code would help in certifying state illness benefit claims.

### Access of secondary care

**Fig. 3:**
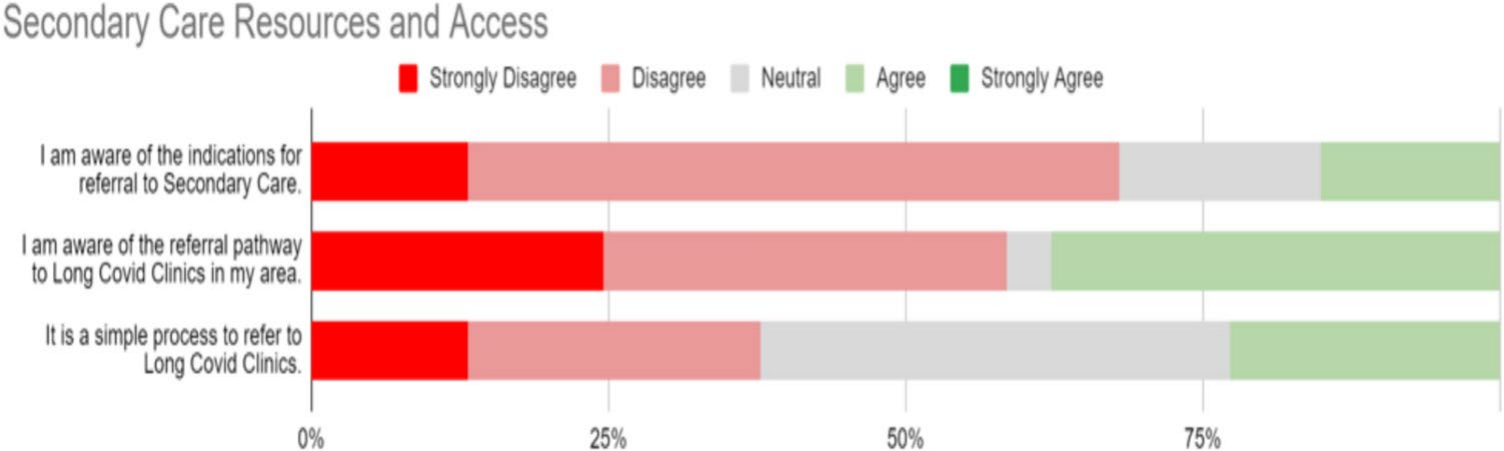
Secondary care resources for Long Covid

Responses to statements regarding Secondary Care Resources for LC are represented in Fig. 3. In response to the statement “I am aware of the indications for referral to secondary care”, 70% (36/53) of responses were either strongly disagree or disagree. Respondents were more likely to agree with the statement that they were “aware of the referral pathways” to LC services in secondary care, with 37.74% (20/53) agreeing, but there were also 58.50% (31/53) strongly disagree or disagree responses. A statement that the referral process is simple generated a mixed response, but no strong agreement with the statement. 43.4% of participants reported that they had referred to a LC secondary care clinic. Free-text responses indicated heterogenous experiences with waiting times and acceptance of referrals. Some reported positive patient feedback concerning these clinics, while others reported that clinic review resulted in no change in patient management. A lack of multi-disciplinary team resources was indicated. Greater accessibility was also reported for private medical clinics.

### Education, training and guideline resources

**Fig. 4:**
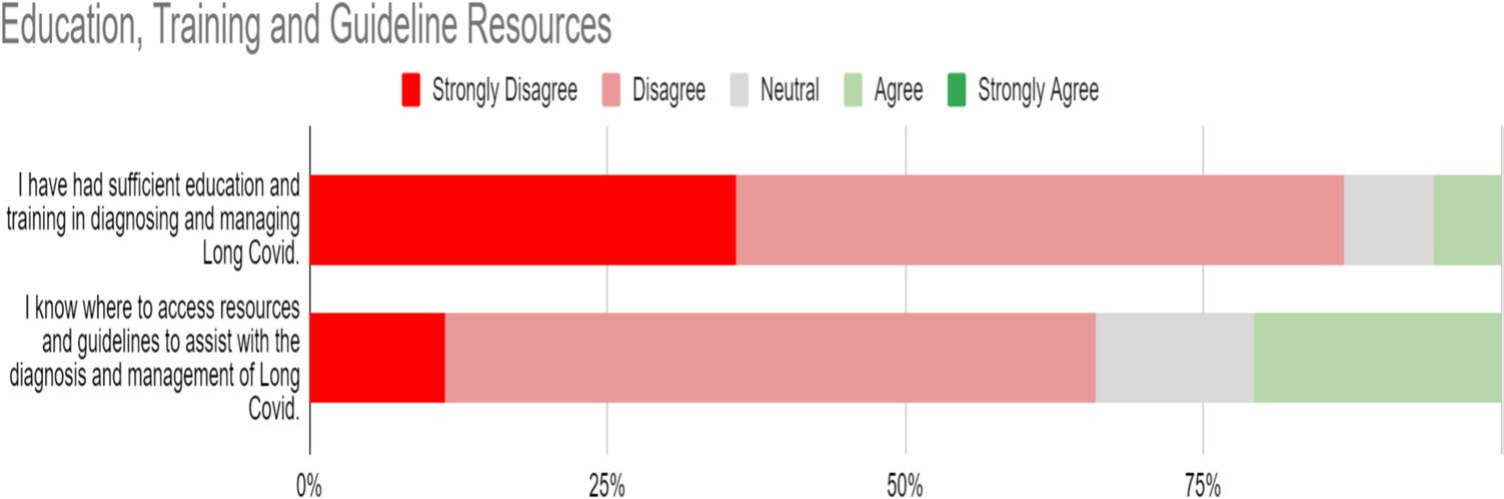
Education, training and guideline resources for Long Covid

Responses to statements regarding Education, Training and Guideline Resources for LC are represented in Fig. 4. The statement GPs disagreed with most in this survey was that they had “had sufficient education and training in diagnosing and managing Long Covid”, with just 5.66% (3/53) agreeing with the statement. Furthermore, 92.45% (49/53) of respondents answered “yes” to the dichotomous statement that there were educational deficits relating to LC. Free text expansions on this response indicated that the educational deficiencies were identified in diagnosis, overall uncertainty about an incipient illness, referral criteria and pathways, educational resources, guideline availability, prognostication, and interventions. 66% (35/53) either strongly disagreed or disagreed with a statement of awareness about access to resources and guidelines.

Of 10 pre-defined resource suggestions, 81.1% (43/53) identified an Irish College of General Practitioner’s Quick Reference Guideline as potentially helpful. In decreasing order, pre-written patient information leaflets, a webinar on the topic, an Irish Health Service Executive information web page were all also identified as potentially helpful by more than half of the respondents.

## Discussion

The aim of this study was to establish GPs experiences with LC across the domains of diagnosis, management, education, and service-related impacts We found broadly low levels of confidence across the domains studied. Overall, responses indicate low confidence in diagnosis and management of LC, as well as navigating the interface with secondary care. Lack of confidence could contribute to often-negative patient experiences [14, 15]. LC can vary in its clinical presentation which is in keeping with the findings of this study; however, a general pattern of clinical features is recognised [5, 22]. We also confirm that cases of LC are being identified in the Irish paediatric population, a group at risk of being overlooked [23].

A 2022 survey of GPs in Germany identified that treatment options for LC were suboptimal [21]. Limited treatment options, therefore, rather than educational or resource deficits alone, may explain why our group demonstrated a corresponding lack of confidence in the management of the condition. Slovenian GPs reported that managing patients absent from work due to LC was particularly challenging, partly due to system-related barriers involving the submission of illness reports [20]. Our study suggests that a LC disease code is considered beneficial in this regard.

We also identified uncertainty in GPs’ awareness of indications for and process of referral to secondary care for LC. Notably, there is an established public clinic treating LC in the region of this study’s enquiry. Private services are also available. This study included reports of discrepancy between the availability of public and private services. This is significant, as there may exist a reinforcing feedback loop between socio-economic deprivation and LC, where disproportionate vulnerability to and economic consequences of LC are observed [20, 24, 25].

Educational, training and guideline resources may be conceived as potentially beneficial given the challenges experienced by GPs, as has previously been proposed [17]. This study would support this conclusion, finding a notable 92.45% of GPs recognised educational deficits regarding the diagnosis of LC. Guideline resources are identified as a key intervention, corresponding with German GPs’ preferences [19].

This study has some limitations. The network of GP training practices included may limit the study’s external validity as it is confined to a certain geographical area. Participants demonstrated a low degree of confidence but a large number of respondents were GPs Registrars who generally have a low degree of confidence in managing many aspects of general practice. The study is also limited by its small sample size and short duration (one month) of data collection.

Future research should be directed towards more detailed qualitative and quantitative examination of GPs’ experience with LC, potentially seeking interventional targets to improve GP confidence with the disease and seeking to identify appropriate educational and training measures. Accessibility of care may require further examination. Ongoing efforts to gather and distribute guideline resources should also be supported.

## Conclusions

This study provides insight into the experiences and challenges faced by GPs in the care of patients with LC. Overall, there was a lack of confidence in the diagnosis and management of LC amongst GPs. Most GPs were unaware of the indications for referral to secondary care or the referral pathways available to them. There is a need for educational tools and supports to assist GPs with their assessment and management of this emerging condition.

## Supplementary Information

Supplementary file1 (DOCX 20.6 KB)
