# Supplementary material for: An investigation into General Practitioners’ experience with Long Covid
Source: Ir J Med Sci. 2024 Aug 20;193(6):2869–73. doi: 10.1007/s11845-024-03782-7 (PMC11666628; doi:10.1007/s11845-024-03782-7)
Supplement: Supplementary file 1 — Supplementary file1 (DOCX 20.6 KB) [file 11845_2024_3782_MOESM1_ESM.pdf]

General Practitioner's Experience of Long Covid – Questionnaire

Please choose one:

1. Qualification:	Qualified GP	GP Trainee	
2. Years experience:	<5 years	5-10 years	>10years
3. Location of work:	Rural Practice	Urban Practice	Mixed
4. Sessions worked per week:	0-3	4-6	7+

5. Is Long Covid relevant to your daily practice? Yes No

6. I have diagnosed and managed Long Covid:
   (a) In paediatric patients Yes No

   Estimated total number of patients: _______________

   (b) In adult patients Yes No

   Estimated total number of patients: _______________

Please tick your level of agreement with each statement below (number 7-16):

	Strongly disagree	***Disagree***	***Neither disagree or agree***	***Agree***	***Strongly agree***
**Assessment and Diagnosis:**					
7. I am confident in diagnosing Long Covid					
8. I am confident of when to arrange investigations for patients presenting with symptoms of possible Long Covid					
**GP Management:**					
9. I feel confident explaining a diagnosis of Long Covid to my patients					

10. I feel confident treating a patient with Long Covid					
11. I am confident in my understanding of the prognosis of Long Covid					
**Secondary Care Resources and Access:**					
12. I am aware of the indications for referral to Secondary Care					
13. I am aware of the referral pathway to Long Covid Clinics in my area					
14. It is a simple process to refer to Long Covid Clinics					
**Education, Training and Guideline Resources:**					
15. I have had sufficient education and training in diagnosing and managing Long Covid					
16. I know where to access resources and guidelines to assist with the diagnosis and management of Long Covid					

Please answer yes or no to the following questions (number 17-20):

17. Do you use certain symptom criteria to diagnose Long Covid?
    Yes No

    If you answered yes, please describe what these criteria are:

    ____

18. Do you think there are educational deficits regarding the diagnosis of Long Covid?
    Yes No

    If you answered yes, please describe what you believe these deficits are:

____

19. Would a ‘Long Covid’ social welfare code assist with your daily practice in completing sick certificates?
    Yes No

20. Have you ever made a referral to a Long Covid Clinic?
    Yes No

    If you answered yes, can you tell us about your experience. (waiting times, response back)

    ____

21. What resources would help you in your daily practice with the diagnosis and management of Long Covid? Pick tick all that apply

CME	
GP Training Curriculum	
Webinar	
GP Fellowship in Long Covid	
Quick Reference Guide	
GP Special Interest Group	
Patient Support Group	
Patient information leaflets	
HSE Conditions Page	
Covid Rehabiliation Group	
Other	

Thank you for completing this questionnaire.
